# Reliable and valid NEWS for Chinese seniors: measuring perceived neighborhood attributes related to walking

**DOI:** 10.1186/1479-5868-7-84

**Published:** 2010-11-25

**Authors:** Ester Cerin, Cindy HP Sit, Man-chin Cheung, Sai-yin Ho, Lok-chun Janet Lee, Wai-man Chan

**Affiliations:** 1 Institute of Human Performance, The University of Hong Kong, 111-113 Pokfulam Rd., Hong Kong SAR; 2 Elderly Health Service, Department of Health, Room 3502-4, 35/F Hopewell Centre, 183 Queen's Road East, Wan Chai, Hong Kong SAR; 3 School of Public Health, The University of Hong Kong, Hong Kong SAR

## Abstract

**Background:**

The effects of the built environment on walking in seniors have not been studied in an Asian context. To examine these effects, valid and reliable measures are needed. The aim of this study was to develop and validate a questionnaire of perceived neighborhood characteristics related to walking appropriate for Chinese seniors (Neighborhood Environment Walkability Scale for Chinese Seniors, NEWS-CS). It was based on the Neighborhood Environment Walkability Scale - Abbreviated (NEWS-A), a validated measure of perceived built environment developed in the USA for adults. A secondary study aim was to establish the generalizability of the NEWS-A to an Asian high-density urban context and a different age group.

**Methods:**

A multidisciplinary panel of experts adapted the original NEWS-A to reflect the built environment of Hong Kong and needs of seniors. The translated instrument was pre-tested on a sample of 50 Chinese-speaking senior residents (65+ years). The final version of the NEWS-CS was interviewer-administered to 484 seniors residing in four selected Hong Kong districts varying in walkability and socio-economic status. Ninety-two participants completed the questionnaire on two separate occasions, 2-3 weeks apart. Test-rest reliability indices were estimated for each item and subscale of the NEWS-CS. Confirmatory factor analysis was used to develop the measurement model of the NEWS-CS and cross-validate that of the NEWS-A.

**Results:**

The final version of the NEWS-CS consisted of 14 subscales and four single items (76 items). Test-retest reliability was moderate to good (ICC > 50 or % agreement > 60) except for four items measuring distance to destinations. The originally-proposed measurement models of the NEWS-A and NEWS-CS required 2-3 theoretically-justifiable modifications to fit the data well.

**Conclusions:**

The NEWS-CS possesses sufficient levels of reliability and factorial validity to be used for measuring perceived neighborhood environment in Chinese seniors. Further work is needed to assess its construct validity and generalizability to other Asian locations. In general, the measurement model of the original NEWS-A was generalizable to this study context, supporting the feasibility of cross-country and age-group comparisons of the effect of the neighborhood environment on walking using the NEWS-A as a tool to measure the perceived built environment.

## Background

The number of senior residents in China is projected to exceed a third of the total population in 2060, approximately corresponding to 500 million [1]. Increases in the proportion of seniors require that special attention be given to their health. There is compelling evidence that an active lifestyle can contribute to health aging [2]. Health-enhancing amounts of physical activity can be accumulated through walking [3], which is one of the recommended forms of physical activity for seniors because it is versatile, affordable, and safe [4].

There is growing, albeit inconsistent, evidence that the neighborhood built environment can facilitate or hinder residents' engagement in walking for different purposes in both adults and seniors [4-9]. Conflicting findings have in part been attributed to using different built-environment measures with sometimes doubtful or unknown metric properties [4,10]. To address this problem, there have been recent calls for the development of comparable and good-quality measures of the built environment [10,11]. The built environment has been assessed using three main methods: questionnaires, systematic observations, and archival data sets layered and analyzed with Geographic Information Systems (GIS). While systematic observations and GIS databases provide objective data on neighborhood attributes, questionnaires gauge residents' perceptions of the local environment. These methods complement each other and offer somewhat different but equally important information contributing to a better understanding of walking behavior [12-15].

Of all available questionnaires assessing perceived attributes of the neighborhood environment believed to impact on residents' walking, the Neighborhood Environment Walkability Scale (NEWS) [16] and its abbreviated version (NEWS-A) [17] are the most frequently used internationally [7,18-20]. Their popularity is in part due to them being the 'official' instruments of the International Physical Activity and the Environment Network, an international collaborative research initiative aimed at conducting cross-country pooled analyses of built-environment and physical activity relationships. The NEWS-A has been translated in many languages and found to be sufficiently reliable and valid in adult populations [16-19,21-23].

However, the NEWS-A was developed in the USA, which raises some concerns about its applicability to geographical locations differing in environmental attributes and culture [18,24]. Asian urban environments are much denser both in terms of population and destinations than those of countries such as the USA and Australia [18]. Additionally, they exhibit walking-related environmental idiosyncrasies (e.g., air pollution, crowdedness, diverse terrain, types of destinations) that are not captured by tools developed for less dense locations. Although the Chinese version of the NEWS-A showed good reliability and construct validity in a sample of Hong Kong adults [18], it does not include items measuring environmental attributes relevant to Asian high-density urban environments and, thus, its value in a geographically-specific context is limited.

Another limitation associated with the NEWS-A is that it was developed and tested on adult populations (20-65 years). Therefore, its suitability for older adults (65+ years) is uncertain. To gain a better understanding of how the built environment affects the physical activity levels of populations and, subsequently, devise effective environmental interventions advantageous to the majority of residents, it is also important to identify age-group differences in effects. This implies the use of measures that are both comparable (e.g., consist of common items) and age specific (i.e., gauge environmental attributes that are of particular relevance to specific age groups). However, although recent studies have used the full version or selected items of the NEWS-A with older adults [7,20], to these authors' knowledge, no studies have yet attempted to adapt and/or conduct reliability and factorial validity tests of the original or adapted versions of the NEWS-A on this particular age group. To address the lack of questionnaires measuring universally-comparable environmental aspects related to walking as well as aspects specific to an Asian urban context and older residents (65+ years), the aim of this study was to develop a new version of the NEWS-A for Chinese senior residents (thereafter termed NEWS-CS) and test its reliability and factorial validity on a sample of Hong Kong seniors.

## Method

This study consisted of three stages: (1) development and translation; (2) pilot study including cognitive testing; (3) reliability and factorial validity testing. The study protocol was approved by the ethics committees of participating research institutions.

### Measures

The original English [17] and Chinese translation [18] of the Neighborhood Environment Walkability Scale - Abbreviated (NEWS-A) were the source instruments used to develop the Neighborhood Environment Walkability Scale for Chinese Seniors (NEWS-CS). The original NEWS-A assesses perceived environmental characteristics, stemming in part from the urban planning literature, believed to influence walking and other forms of physical activity [16,17]. It consists of 54 items. These are grouped into subscales including perceived residential density, proximity to non-residential land uses (land use mix - diversity), ease of access to non-residential uses (land use mix - access), street connectivity, infrastructure for walking and cycling, aesthetics, traffic safety and safety from crime. Four single items assess hilly streets, difficult car parking in shopping areas, absence of cul-de-sacs, and perceived major physical barriers to walking. All subscales and single items, with the exception of residential density and land use mix - diversity, are rated on a 4-point Likert scale. Residential density items use a 5-point scale, whereby ratings are weighted relative to the average residential density that a specific item represents. The weighted ratings are summed to create a perceived residential density score. Land use mix - diversity is assessed by the perceived walking proximity from home to various destinations, with responses ranging from 1- to 5-minute to >30-min walking distance. The NEWS-A has been shown to have acceptable levels of validity and reliability [16-18]. A study on Hong Kong adults reported high levels of test-retest reliability of the Chinese version of the NEWS-A [18].

The version of the NEWS-CS examined in this study encompasses all but two items of the NEWS-A, in their original or slightly modified form, and 24 additional items describing features of the environment relevant to the study setting and senior residents (see Development and translation section below).

### Participants and procedures

#### Development and translation

A multi-disciplinary panel of experts from the fields of public health (n = 3), urban planning (n = 1), Elderly Health Service (n = 4), physiotherapy (n = 1) and physical activity (n = 3) reviewed the original English and Chinese versions of the NEWS-A, and adapted them to reflect the built environment of Hong Kong and needs of older adults [25]. The panel's adaptation of the NEWS-A (thereafter, NEWS-CS) included all items of the original instrument, in their original or slightly modified form, except for one (see Table 1). In total, 23 items describing features of the environment relevant to the study setting and target population were added and 13 were modified.

**Table 1:** Adaptations of the NEWS-A for Chinese older adults

Item	Reason
*Additions*	
Distance to doctor/clinical service ^a^	Relevant to older adults (health issues)
Distance to Chinese coffee/tea or noodle shop ^a^	To distinguish Chinese-style from Western cafés; differences in affordability and food
Distance to Chinese non-fast food restaurant ^a^	To distinguish Chinese-style from Western restaurants; differences in affordability and food
Distance to community or elderly centre ^a^	Relevant to older adults (place for socializing)
Distance to places of worship (church, temple) ^a^	Relevant to older adults (common practice)
Distance to public toilet ^a^	Relevant to older adults (incontinence)
Distance to bakery/cake shop ^a^	Common food outlet in Hong Kong
Distance to betting branch ^a^	Popular activity among Hong Kong residents
Shopping areas are easily accessible via public transport ^a^	The public transport network in Hong Kong is very developed. This facilitates access to services outside the neighborhood.
The streets are so crowded that it is difficult to walk ^a^	Characteristic of many urban areas of Hong Kong
Need to walk over a bridge or through a tunnel to access the nearest services ^a^	Common feature of Hong Kong.
Can easily access the entrance/exit of the building I live in (e.g., there is a lift that I can use) ^a^	Lack of lifts in high-rise buildings poses accessibility problems to older adults
There are 'hawkers' and shops on the streets and sidewalks blocking the way ^a^.	Common obstacle on Hong Kong streets.
There are many covered sidewalks in my neighborhood. ^a^	Common feature of Hong Kong. Facilitates walking in wet or hot weather conditions.
There are indoor, air-conditioned places (shopping malls) where people can walk ^a^	Common feature of Hong Kong. Facilitates walking in wet or hot weather conditions.
The streets of my neighborhood are often slippery. ^a^	Hazard on steep road sections, common in Hong Kong.
There are sitting facilities (e.g., benches) where I can rest in my neighborhood ^a^	Important feature for older adults
The level of air pollution in my neighborhood is often high ^a^	Air pollution, especially road-side pollution levels, can be very high.
There are lots of animal droppings making walking unpleasant ^a^.	Some areas of Hong Kong have high rates of dog ownership. This creates some street hygiene problems, especially unpleasant in warmer weather.
It is unsafe to walk in my neighborhood because of objects dropping from high-rise buildings ^a^	A common problem in some areas of Hong Kong.
There are parked vehicles that block my vision and make it difficult to safely cross the road ^a^	Common problem in older adults, less capable of quickly reacting to traffic conditions
I am afraid to cross the road because there are too many passing cars ^a^	Common problem in older adults, less capable of quickly reacting to the traffic conditions
There are many homeless people, drug addicts and/or prostitutes in my neighborhood ^a^	Signs of social disorder are more common than actual crime, which is very low in Hong Kong.
It would be difficult to ask for help in my neighborhood because there are not many people around ^a^	Important issue for older fragile adults.
*Modifications*	
Two items measuring commonness of detached single-family residences and townhouses or row hoses of 1-3 stories merged into a single item ^a^	Uncommon type of housing in Hong Kong urban areas
Item measuring commonness of apartments of more than 13 stories changed to 'apartments of 13-20' stories. Additional item referring to commonness of apartment buildings with more than 20 stories added ^a^	A large percentage of residential buildings have more than 20 stories.
Destination 'fruit/vegetable market' renamed 'fresh food market' ^a^	Markets usually sell vegetables, fruit as well as fresh meat and seafood
Destination 'other schools' changed to 'nursery schools' ^a^	It is common for Hong Kong older adults to drop and pick up their grandchildren
Destinations 'fast-food restaurant' renamed 'Chained Western or Chinese fast-food restaurant' ^a^	Clarify type of destination to respondents. Western and Chinese fast-food restaurants sell similarly priced products
Destination 'non-fast food restaurant' changed to 'Western/international non-fast food restaurant' ^a^	To distinguish Chinese-style from Western restaurants; differences in affordability and food
Destination 'recreation centre' changed to 'swimming pool' ^a^	Swimming is a very popular recreational activity among Hong Kong older adults
Destination 'clothing store' changed to 'clothing & shoe store' ^a^	Linguistically more appropriate
Item 'Parking is difficult in local shopping areas' omitted ^b^	Not relevant because they do not drive nor own a car
Destination 'your job or school' ^b^	Not relevant as they are retired
'Sidewalks are separated from the road/traffic by parked cars' changed to 'there are motor vehicles parked on the sidewalks making it difficult to walk' ^b^	Cannot understand the purpose of the original item. Parked cars relevant features only if perceived as an obstacle to walking.
'There is a grass/dirt strip that separates the streets from the sidewalks' changed to 'there is a fence that separates the streets from the sidewalks' ^a^	Grass/dirt strips a very uncommon in Hong Kong. Metal fences are used to separate sidewalks from roads.
'Walkers and bikers on the streets can be easily seen by people in their homes' changed to 'walkers on the streets can be easily seen by other people' ^a^	Bikers are rare n Hong Kong. Most residences are in high-rise buildings making it difficult to see walkers on the streets. There are usually other people on the streets.

^a ^Generated by expert panel; ^b ^Generated by respondents.

Specifically, the 'residential density' subscale was modified to capture the high level of residential density typical of Hong Kong. Eight types of destinations were added to the 'land use mix - diversity' subscale. The descriptors of six other destinations were modified to provide a better match to either the local environment or senior residents (Table 1). Modifications were also proposed to three original items measuring infrastructure and safety for walking. Eleven items were added to other subscales of the NEWS-A in order to capture particular features of the built environment characteristic of Hong Kong - namely, a highly developed public transport network, high levels of population density and crowdedness, terrain steepness, air pollution, indoor-places for walking, safety issues related to high-rise buildings, street hygiene concerns associated with to the presence of dogs, and aspects of social disorder (Table 1). Five newly-developed items were included to gauge aspects of the environment believed to be particularly important to senior residents (e.g., sitting facilities, road and traffic hazards, access to the entrance and exit of high-rise buildings, ability to ask for assistance) [25].

The proposed response scales for the 'residential density' and land use mix - diversity' subscales were the same as those in the original NEWS-A. In addition to the original response scale (*strongly disagree*, *somewhat disagree*, *somewhat agree*, *strongly agree*), two additional Likert-like scales were proposed for the remaining items of the NEWS-CS. Two panel members maintained that the respondents might find the word 'somewhat' confusing. Therefore, the use of a scale with the response anchors *strongly disagree*, *disagree*, *agree*, *strongly agree *was suggested. Others suggested that the response anchors *disagree*, *somewhat disagree*, *somewhat agree*, and *agree *might be more appropriate since previous research had shown that some Asian populations (China and Japan) tend to avoid endorsing extreme answers [26]. Such tendency can have a negative effect on response variability and, hence, the ability to evaluate relationships between variables [26].

The English versions of the NEWS-CS with three different response scales were translated into Chinese following the World Health Organization guidelines [27]. This included (1) forward independent translations by two bilingual Chinese native speakers familiar with the areas of study (physical activity and urban planning); (2) an assessment of the accuracy of the translations by two independent bilingual experts in survey development; (3) discussion of translation problems and consensual amendments to the forward translation by the panel of experts in collaboration with the translators; (4) a back-translation of the NEWS-CS into English by an independent translator with no knowledge of the original questionnaire. The last three steps were reiterated until equivalence between the original English version of the NEWS-CS and the back-translation of the Chinese NEWS-CS was achieved.

#### Pilot testing and cognitive interviews

The translated instruments were pre-tested on 50 Chinese-speaking persons aged 65 or above, able to walk without assistance and communicate verbally, with no diagnosis of cognitive impairment and residing in the community (Table 2). Participants were selected and screened for eligibility from membership lists of four Elderly Health Centres (EHCs), representing areas of Hong Kong differing in socio-economic status (SES) and transport-related walkability (see below for details) [28]. The EHCs, one in each of the 18 districts of Hong Kong, were established by the Department of Health of the Government of the Hong Kong Special Administrative Region to provide comprehensive primary care services for elderly aged 65 and over residing in Hong Kong on a membership basis. It was decided to sample participants from the EHCs because of their appropriate age, observed willingness to participate in health-related studies, the ability to pre-screen their health with the assistance of EHCs staff, and their similarity in age, SES, and health status to the general elderly population of Hong Kong [29]. EHC members differ from the general population in gender distribution (66% vs. 54% women) and education level in men only (slightly higher education level among EHC members) [29].

**Table 2:** Socio-demographic and area characteristics of study samples

Characteristic	Pilot study		Main study	
	N	%	N	%
*Participants*				
Gender	25	50	201	42
Male	25	50	283	58
Female				
Age				
65 - 74 years	28	56	324	67
75 - 84 years	18	36	150	31
85+ years	4	8	10	2
Educational attainment				
Secondary or above	21	42	189	39
Primary	23	46	232	48
No formal education but can read and write	7	14	49	10
Illiterate	0	0	14	3

*Study areas (districts)*	HWHSES	LWHSES	HWLSES	LWLSES

Number of participants in pilot study	12	14	12	12
Number of participants in main study	120	120	124	120
District median monthly household income (HK$) ^b^	25,000	20,000	12,200	13,000
% of owner occupiers in district ^b^	62	62	42	50
Intersection density (intersections/km^2^)	165	15^a^	197	12^a^
Household density (number of households/km^2^)	5861	2514^a^	12216	1979^a^
Commercial/service destination density (units/km^2^) ^d^	1643	72^a^	1584	88^a^

^a ^park land area excluded; ^b ^Data supplied by the Census and Statistics Department of Hong Kong SAR (2006); Computed using data from Centamap www.centamap.com; ^d ^Computed using data from the Quarterly Survey of Employment and Vacancies (Census and Statistics Department of Hong Kong SAR, March, 2006). Data on two categories of establishments relevant to residents' transport-related walking were included: (1) community, social and personal services; (2) Wholesale, retail and import and export trades, restaurants and hotels; HW = high walkable; HSES = high socio-economic status; LW = low walkable; LSES = low socio-economic status.

Participants were balanced by gender and EHCs (Table 2). After signing an informed consent form, they were randomized to one of the three versions of the NEWS-CS (with different response scales) and asked to verbalize their thought process while they were providing responses to the items. They were also asked questions about the meaning, the choice of words and appropriateness of each item in the questionnaire. Additionally, they were invited to reveal any aspect of the built environment relevant to their walking that was not included in the NEWS-CS. The results of the pilot study were used to subsequently modify the NEWS-CS.

#### Reliability and factorial validity testing

A sample of 484 of eligible seniors (able to walk without assistance, with no diagnosis of cognitive impairment and residing in one of a priori selected locations) were recruited from membership lists of four EHCs selected based on available data on walkability and SES of their catchment areas (districts) to represent the following strata: high walkable/high SES; high walkable/low SES; low walkable/high SES; and low walkable/low SES (Table 2). This type of stratification was needed to maximize variance in the environmental variables of interest. Median household income and percentage of owner-occupiers were used as measures of area SES and were obtained from the Census and Statistics Department of Hong Kong SAR. Household density (number of households per km^2^), intersection density (number of intersections with three or more unique intersecting streets per km^2^) and commercial and service destination density (commercial and service destinations per km^2^) were used as measures of walkability. This information was obtained from the Census and Statistics Department and from Centamap (http://www.centamap.com; Table 2).

Eight street blocks, with at least 25 EHCs members residing in the block, were randomly selected in each area (total 32 street blocks). Approximately 15 EHCs members were recruited from each street block. The NEWS-CS was interviewer-administered to consenting participants (recruitment rate: 78% of the total number of invited participants). Women [χ^2^(1) = 3.90; *p *= .048] and elders living in lower SES areas [χ^2^(1) = 5.73; *p *= .017] were slightly more likely to consent to participation. Socio-demographic information was also collected (age, gender, and educational attainment). Completion of the NEWS-CS and socio-demographic questions took, on average, ~25 minutes. All participants were asked whether they would be willing to be re-assessed two weeks after the first assessment. Three consenting participants per street block were recruited for this component of the study (n = 96). Ninety-two participants attended both interviews. The interval between the two interviews ranged from 14 to 20 days (average of 17 days).

### Data analytic plan

#### Assessment of Likert scales (pilot study)

To examine whether the Likert scale used in the original NEWS-A would result in a potential reduction of variability in responses due to the tendency to avoid endorsing extreme responses (i.e., strongly agree), standard deviations of the scores were computed for each relevant NEWS-CS item by type of Likert scale (three types were used), and entered as data in the analyses. The items represented cases, while the three types of Likert scale represented conditions (i.e., independent variables). Given that the distribution of standard deviations was positively skewed, the Wilcoxon signed-rank test was used to test the significance of the difference between the standard deviations obtained using different Likert scales.

#### Test-retest reliability (main study)

Test-retest reliability of each item and subscale of the NEWS-CS was established by computing intraclass correlation coefficients. Based on previously proposed classification systems, ICC values below 0.50 were classified as poor, 0.50 to 0.75 as moderate, and above 0.75 as high levels of reliability [30]. Percentage agreement was also computed as an additional measure of test-retest reliability to assess the percentage of individuals who gave the same response on an item on both assessments. Percent agreement was also computed for subscales with restricted variability (SD < 0.5), as low levels of variability may result in very low ICC values even though the actual agreement between assessment is high. Items and subscales with moderate or low ICC values, restricted variability (SD < 0.5), but percent agreement greater than 75% were considered highly reliable. While items and scales with low ICC, but percent agreement exceeding 60%, were considered moderately reliable [31].

#### Factorial structure (main study)

Confirmatory factor analysis (CFA) was performed on the responses of the NEWS-CS for items that could be factor-analyzed (i.e., all items but those measuring perceived dwelling density and land use mix diversity). To account for street-block clustering effects threatening the validity of the standard errors estimates, CFAs were conducted using the within-neighborhood variance/covariance matrix, containing estimates of relationships among differences in responses between participants living in the same street-blocks [17,21]. Multilevel CFA, simultaneously modeling within- and between-street-block covariances, could not be employed since the number of sampled street blocks (32) was too small for the number of factor-analyzable items (40). CFA was conducted using the Maximum Likelihood Estimation (MLE) method. Based on previous findings [17,19,21] and a preliminary classification of newly-added items, a measurement model of the NEWS-CS including the following factors and single items was estimated: access to services (4 items); street connectivity (2 items); infrastructure for walking (4 items); indoor places for walking (2 items); physical and social disorder (3 items); aesthetics (4 items); crowdedness (2 items); presence of people (2 items); traffic and road hazards (8 items); crime (3 items); hilly streets (single item); physical barriers to walking (single item); dead ends (single item); easy access to entrance of residence (single item); sitting facilities (single item); bridge or tunnel (single item). Another model determined whether the factor structure of the original NEWS-A estimated on a sample of American adults [17] held for the examined sample of Chinese older adults. This model was estimated using only factor-analyzable items common to the NEWS-A and NEWS-CS (23 items). It consisted of the following factors and single items: access to services (3 items); street connectivity (2 items); infrastructure for walking (5 items); aesthetics (4 items); traffic hazards (3 items); crime (3 items); hilly streets (single item); physical barriers to walking (single item); dead ends (single item). The models were re-specified following the iterative model-generating approach outlined by Jöreskog and Sörbom [32]. Model re-specification was based on an analysis of standardized factor loadings, standardized residual covariances, Wald tests, univariate Lagrange multiplier tests, and substantive considerations. Factor loadings with an absolute value greater than 0.30 were considered to be significant. A combination of indices of model fit, as proposed by Hu and Bentler [33], and Kline [34], was used to assess the goodness-of-fit of the measurement models. We used the standardized root mean squared residual (SRMS), the root mean square error of approximation (RMSEA), and the comparative fit index (CFI). Values indicative of good model fit are ≤0.08 for SRMR, ≤0.06 for RMSEA, and ≥0.95 for CFI [33]. We also reported the χ^2 ^test and the Akaike information criterion (AIC) of the standard goodness-of-fit χ^2 ^statistic including a penalty for complexity. CFAs were conducted using EQS 6.1 (Multivariate Software Inc., Encino, CA, 2004).

## Results

### Assessment of Likert scales (pilot study)

One of the aims of the pilot study was to examine whether the use of different anchors with a 4-point Likert scale would result in significantly different levels of variability in responses. Standard deviations of scores on items rated using the original Likert scale (*strongly agree*, *somewhat agree*, *somewhat disagree*, *strongly disagree*) were significantly larger (*M *= 0.92; Median = 1.10) than those obtained using a Likert scale without the adjective 'somewhat' (*M *= 0.67; Median = 1.01) (signrank *z *= 3.33; *p *< .001). The Likert scale without the adjective 'strongly' yielded similar standard deviations (*M *= 0.96; Median = 1.12) to those of the original Likert scale (signrank *z *= -1.63; *p *= .103) but larger standard deviations than the scale without the adjective 'somewhat' (signrank *z *= -4.59; p < .001). Given that the original Likert scale yielded one of the largest levels of variability in responses, it was used in the main study.

#### Test-retest reliability (main study)

Table 3 shows the descriptive statistics and test-retest reliability indices for each item and subscale of the NEWS-CS. In general, the reliability of the subscales was moderate, with ICCs ranging from 0.52 to 0.77. The subscale 'presence of people' had a low ICC (0.37) but a high percent agreement (87%) indicative of good reliability. These discrepant results were likely due to the low variability in responses (SD = 0.3). Residential density and land use mix diversity were the subscales with the highest reliability indices. At the item level, reliability ranged from poor (e.g., distance to Western non-fast food restaurant) to excellent (e.g., some items measuring residential density) and, in the main, was moderate. Six items had very low ICCs but high percent agreement (e.g., shopping accessible via public transport). All of these items showed low variability in responses, explaining the differences in results. Items with consistently low reliability were distance to 'video/audio shop', 'Western non-fast food restaurant', 'Western coffee shop', and 'public toilet'. Exclusion of these items from the land use mix - diversity scale had minimal impact on the reliability of the subscale (Table 3).

**Table 3:** Descriptive statistics and test-retest reliability of the NEWS-CS (reliability subsample; N = 92)

Items/scale	M (SD) time 1	M (SD) time 2	Agreement (%)	ICC
*A. Residential density*	678 (110)	672 (100)	NA	0.72^a^
1) Detached single-family houses	1.0 (0.3)	1.0 (0.3)	99	0.92
2) Multi-family houses (1-3 stories)	1.3 (0.8)	1.2 (0.5)	85	0.61
3) Apartments with 4-6 stories	1.4 (0.8)	1.3 (0.8)	76	0.63
4) Apartments with 7-12 stories	1.8 (1.1)	1.7 (1.1)	69	0.75
5) Apartments with 13-20 stories	2.1 (1.3)	1.9 (1.2)	63	0.54
6) Apartments with >20 stories	3.9 (1.4)	4.1 (1.3)	67	0.78
*B. Land use diversity (distance to destinations)*	2.1 (0.6) 2.0 (0.6)	2.2 (0.6) 2.2 (0.5)	NA	0.76^b#^ 0.77^b§^
1) Convenience store	1.4 (0.7)	1.5 (0.8)	65	0.53
2) Supermarket	1.6 (0.8)	1.5 (0.7)	55	0.56
3) Fresh-food market	1.8 (0.8)	2.0 (0.9)	73	0.60
4) Hardware store	1.8 (1.3)	1.7 (1.2)	62	0.60
5) Clothing/shoe shop	1.9 (1.3)	2.0 (1.3)	64	0.62
6) Pharmacy/drug store	1.6 (0.9)	1.6 (0.8)	64	0.56
7) Book/stationary shop	2.3 (1.6)	2.5 (1.8)	54	0.59
8) Video/audio shop	2.5 (1.8)	2.3 (1.7)	38	0.50
9) Library	3.4 (1.6)	3.5 (1.6)	60	0.62
10) Laundry/dry cleaner	1.6 (1.1)	1.7 (1.1)	61	0.61
11) Salon/barber shop	1.5 (0.8)	1.3 (1.0)	54	0.51
12) Bank/credit union	2.2 (1.3)	2.2 (1.2)	60	0.72
13) Post office	2.4 (1.3)	2.6 (1.4)	64	0.64
14) Doctor/clinical service	1.7 (0.9)	1.5 (1.0)	60	0.60
15) Primary school	2.1 (1.5)	2.2 (1.5)	68	0.78
16) Nursery school	1.9 (1.4)	1.9 (1.4)	67	0.73
17) Chained Western or Chinese fast food restaurant	1.7 (1.0)	1.5 (1.0)	61	0.53
18) Chinese coffee shop or noodle shop	1.6 (0.9)	1.7 (1.0)	63	0.53
19) Chinese non-fast food restaurant	1.7 (0.8)	1.9 (1.1)	68	0.55
20) Western non-fast food restaurant	2.1 (1.5)	2.5 (1.7)	34	0.25
21) Western coffee shop	2.9 (2.0)	2.7 (2.0)	51	0.37
22) Park	2.0 (1.2)	1.9 (1.1)	62	0.51
23) Community center or elderly center	2.1 (1.3)	2.2 (1.4)	66	0.60
24) Gym or fitness/recreational facility	3.8 (1.8)	3.7 (1.7)	58	0.64
25) Swimming pool	3.7 (2.0)	3.7 (1.9)	65	0.74
26) Religious places	3.1 (2.0)	3.1 (1.9)	61	0.60
27) Public toilet	1.8 (1.3)	2.0 (1.2)	50	0.29
28) Bakery/cake shop	1.4 (0.6)	1.5 (0.7)	67	0.53
29) Public transit	1.4 (0.7)	1.4 (0.7)	62	0.50
30) Hong Kong Jockey Club betting branch	2.9 (1.6)	2.8 (1.6)	78	0.74
*C. Access to services*	3.9 (0.3)	4.0 (0.2)	76	0.62^b^
1) Shops are within walking distance	3.9 (0.4)	3.9 (0.4)	89	0.64
2) Shopping accessible via public transport	3.9 (0.4)	4.0 (0.1)	88	0.28
3) Many places within walking distance	3.8 (0.7)	3.9 (0.4)	84	0.62
4) Easy to walk to transit stop	3.9 (0.3)	4.0 (0.1)	95	0.27
*D. Physical barriers to walking*	1.3 (0.5)	1.2 (0.4)	61	0.57^b^
1) Hilly streets	1.3 (0.8)	1.2 (0.7)	80	0.59
2) Major barriers (e.g., roadwork, freeways)	1.4 (0.8)	1.2 (0.8)	73	0.55
3) Many dead ends	1.2 (0.5)	1.2 (0.6)	86	0.52
*E. Street connectivity*	3.8 (0.4)	3.9 (0.3)	70	0.58^b^
1) Short distance between intersections	3.7 (0.6)	3.8 (0.5)	75	0.52
2) Many alternative routes	3.8 (0.5)	3.9 (0.3)	85	0.57
*F. Infrastructure for walking*	3.9 (0.3)	3.9 (0.2)	76	0.53^b^
1) Sidewalks	3.9 (0.4)	4.0 (0.1)	94	0.41
2) Streets lit at night	3.9 (0.5)	3.9 (0.4)	92	0.65
3) Crosswalks and pedestrian signals to help walkers cross busy streets	3.8 (0.6)	3.8 (0.6)	86	0.51
*G. Indoor places for walking*	2.7 (1.0)	2.9 (1.2)	NA	0.66^b^
1) Many covered sidewalks	2.5 (1.3)	2.6 (1.4)	61	0.70
2) Indoor, air-conditioned places for walking	2.9 (1.3)	3.1 (1.3)	65	0.53
*H. Aesthetics*	2.1 (0.7)	2.0 (0.6)	NA	0.62^b^
1) Trees	3.3 (1.0)	3.4 (1.0)	79	0.63
2) Many interesting things to look at	1.6 (1.0)	1.5 (1.0)	68	0.58
3) Many attractive natural sights	2.1 (1.2)	2.0 (1.3)	75	0.55
4) Attractive buildings/homes	1.6 (1.0)	1.5 (0.9)	67	0.52
*I. Presence of people*	3.8 (0.4)	3.9 (0.3)	87	0.37^b^
1) Walkers can be seen	3.9 (0.3)	4.0 (0.2)	91	0.31
2) Difficult to ask for help because not many people around	1.4 (0.8)	1.3 (0.6)	71	0.50
*J. Crowdedness*	1.6 (0.7)	1.4 (0.8)	NA	0.72^b^
1) Crowded streets	1.6 (1.0)	1.4 (1.0)	70	0.63
2) 'Hawkers' and shops on streets blocking the way	1.5 (0.9)	1.4 (0.9)	79	0.64
*K. Traffic and road hazards*	1.6 (0.6)	1.6 (0.6)	NA	0.62^b^
1) Motor vehicles parked on sidewalks	1.3 (0.5)	1.3 (0.5)	80	0.54
2) Streets and sidewalks slippery	1.3 (0.5)	1.1 (0.4)	87	0.42
3) Air pollution (e.g., exhaust fumes)	1.9 (1.1)	1.8 (1.1)	67	0.58
4) Heavy traffic along nearby streets	1.5 (0.9)	1.6 (1.1)	71	0.53
5) Parked vehicles block vision	1.8 (1.1)	1.7 (1.1)	65	0.70
6) Afraid to cross road because too many passing cars	1.5 (1.0)	1.5 (1.1)	74	0.57
*L. Traffic speed*	1.9 (0.8)	1.6 (0.7)	NA	0.53^b^
1) Slow traffic speed on streets	2.8 (1.1)	3.0 (1.1)	40	0.52
2) Speeding drivers	1.4 (1.0)	1.2 (0.6)	60	0.54
*M. Social disorder/littering*	1.8 (0.7)	1.6 (0.7)	NA	0.62^b^
1) Objects dropping from high-rise buildings	1.9 (1.1)	1.7 (1.1)	65	0.65
2) Animal droppings	2.0 (1.2)	1.8 (1.2)	65	0.51
3) Many homeless people, drug addicts, prostitutes	1.4 (0.8)	1.4 (0.9)	76	0.57
*N. Crime*	1.2 (0.4)	1.1 (0.3)	72	0.62^b^
1) High crime rate	1.1 (0.3)	1.1 (0.3)	73	0.41
2) Unsafe to walk during the day	1.3 (0.7)	1.3 (0.6)	94	0.55
3) Unsafe to walk at night	1.4 (0.8)	1.4 (0.9)	77	0.57
SINGLE ITEMS				
N. *Fence separating sidewalk and traffic*	3.2 (1.1)	3.5 (1.1)	61	0.61
O. *Bridge/overpass connecting to services*	2.1 (1.2)	2.0 (1.3)	65	0.58
P. *Easy access of residential entrance*	3.9 (0.6)	3.9 (0.6)	88	0.68
Q. *Sitting facilities*	2.9 (1.1)	3.0 (1.3)	68	0.61

^a ^computed as (item A1)+(item A2)*12+(item A3)*25+(item A4)*50+(item A5)*75+(item A6)*100; ^b ^average score on items of the subscale (items reverse scored, when appropriate); ^# ^items with poor test-retest reliability (B8, B20, B21, and B27) included; ^§ ^items with poor test-retest reliability (B8, B20, B21, and B27) excluded; grouping of items (subscales) based on the results of the confirmatory factor analyses (see Table 4).

#### Factorial structure (main study)

The originally proposed measurement model of the NEWS-CS met only one of the three goodness-of-fit criteria (CFI = 0.86; SRMR = 0.073; RMSEA = 0.051, 95% CI: 0.048, 0.055; χ2(701) = 1594.7; AIC = 192.7). Standardized loadings were below the criterion value of |.30| for the items 'fence separating sidewalk and traffic', 'slow traffic speed on streets', and 'speeding drivers'. Analysis of indices of poor model fit at the item level lead to several modifications of the measurement model. Firstly the item 'fence separating sidewalk and traffic' was defined as a single item as it did not significantly load on any of the factors. The items 'slow traffic speed on streets' and 'speeding drivers' formed a 'Traffic speed' factor separate from other traffic-related items. The items 'hilly streets', 'many dead ends', and 'major barriers (e.g., roadwork, freeways)' were inter-correlated and significantly loaded on an additional factor named 'Physical barriers to walking' (Table 4). Some factors were not significantly correlated and, thus, their covariance was constrained to zero (Table 5). The model fit of the re-specified model was considerably better than the original model, with two of the three indices meeting the goodness-of-fit criteria and one index meeting a less stringent criterion (CFI = 0.91; SRMR = 0.058; RMSEA = 0.041, 95% CI: 0.037, 0.045; χ2(701) = 1277.5; AIC = -124.5) [34]. All standardized loadings were significant at the 0.001 level. Moderate-to-strong positive correlations were found among 'Traffic and road hazards', 'Crowdedness', and 'Social disorder - littering' (Table 5). The factor 'Indoor places for walking' was substantially negatively related to 'Traffic and road hazards' and 'Physical barriers to walking'. A moderate negative association was also observed between 'Physical barriers to walking' and 'Access to services'.

**Table 4:** Measurement model of the full NEWS-CS and NEWS-CS including items common to the original NEWS-A

	NEWS-CS		NEWS-A	
**Item**	**SL (SU)**	**Factor**	**SL (SU)**	**Factor**

Shops are within walking distance	0.66 (0.56)	Access to services	0.67 (0.55)	Access to services
Shopping accessible via public transport	0.69 (0.53)	"	NA (NA)	NA
Many places within walking distance	0.79 (0.37)	"	0.83 (0.31)	Access to services
Easy to walk to transit stop	0.60 (0.64)	"	0.54 (0.71)	"
Hilly streets	0.72 (0.48)	Physical barriers to walking	0.73 (0.47)	Physical barriers to walking
Major barriers (e.g., roadwork, freeways)	0.59 (0.65)	"	0.63 (0.60)	"
Many dead ends	0.38 (0.85)	"	0.38 (0.85)	"
Short distance between intersections	0.99 (0.02)	Street connectivity	0.99 (0.02)	Street connectivity
Many alternative routes	0.79 (0.38)	"	0.79 (0.38)	"
Sidewalks	0.43 (0.81)	Infrastructure for walking	0.39 (0.85)	Infrastructure for walking
Streets lit at night	0.41 (0.83)	"	0.41 (0.83)	"
Crosswalks and pedestrian signals to help walkers cross busy streets	0.36 (0.87)	"	0.32 (0.90)	"
Many covered sidewalks	0.41 (0.83)	Indoor places for walking	NA (NA)	NA
Indoor, air-conditioned places for walking	0.55 (0.70)	"	NA (NA)	NA
Trees	0.82 (0.33)	Aesthetics	0.79 (0.38)	Aesthetics
Many interesting things to look at	0.41 (0.83)	"	0.42 (0.83)	"
Many attractive natural sights	0.54 (0.71)	"	0.56 (0.69)	"
Attractive buildings/homes	0.46 (0.79)	"	0.48 (0.77)	"
Walkers can be seen	0.57 (0.68)	Presence of people	0.32 (0.90)	Infrastructure for walking
Difficult to ask for help because not many people around	-0.83 (0.32)	"	NA (NA)	NA
Crowded streets	0.56 (0.68)	Crowdedness	NA (NA)	NA
'Hawkers' and shops on streets blocking the way	0.44 (0.81)	"	NA (NA)	NA
Motor vehicles parked on sidewalks	0.50 (0.75)	Traffic and road hazards	NA (NA)	NA
Streets and sidewalks slippery	0.38 (0.86)	"	NA (NA)	NA
Air pollution (e.g., exhaust fumes)	0.61 (0.63)	"	NA (NA)	NA
Heavy traffic along nearby streets	0.60 (0.64)	"	0.30 (0.91)	Traffic load
Parked vehicles block vision	0.51 (0.74)	"	NA (NA)	NA
Afraid to cross road because too many passing cars	0.58 (0.66)	"	NA (NA)	NA
Slow traffic speed on streets	-0.49 (0.77)	Traffic speed	-0.58 (0.66)	Traffic load
Speeding drivers	0.99 (0.02)	"	0.80 (0.36)	"
Objects dropping from high-rise buildings	0.53 (0.72)	Social disorder/littering	NA (NA)	NA
Animal droppings	0.51 (0.74)	"	NA (NA)	NA
Many homeless people, drug addicts, prostitutes	0.45 (0.80)	"	NA (NA)	NA
High crime rate	0.60 (0.64)	Crime	0.60 (0.64)	Crime
Unsafe to walk during the day	0.60 (0.64)	"	0.60 (0.64)	"
Unsafe to walk at night	0.83 (0.32)	"	0.84 (0.71)	"
Fence separating sidewalk and traffic	- (1.00)	Single item	- (1.00)	Single item
Bridge/overpass connecting to services	- (1.00)	"	NA (NA)	NA
Easy access of residential entrance	- (1.00)	"	NA (NA)	NA
Sitting facilities	- (1.00)	"	NA (NA)	NA

NA = not applicable; SL = standardized factor loadings; SU = standardized uniquenesses

**Table 5:** Correlations between latent factors of the full NEWS-CS (above diagonal) and NEWS-CS including only items common to the NEWS-A (below diagonal)

Factors		PBW	SC	IW	IPW	A	PP	CD	TRH	TS	SDL	CR	Factors
		-.45	.12	.35	.32	< |.10|^a^	.24	< |.10|^a^	< |.10|^a^	< |.10|^a^	< |.10|^a^	< |.10|^a^	AS
			< |.10|^a^	-.31	-.70	< |.10|^a^	-.12	.21	.30	< |.10|^a^	.35	< |.10|^a^	PBW
				< |.10|^a^	.13	< |.10|^a^	< |.10|^a^	.11	< |.10|^a^	< |.10|^a^	< |.10|^a^	< |.10|^a^	SC
					.39	< |.10|^a^	.30	< |.10|^a^	-.32	< |.10|^a^	< |.10|^a^	-.32	IW
						.37	.24	-.24	-.46	-.20	-.35	-.25	IPW
PBW	-.49						.37	-.20	-.12	< |.10|^a^	< |.10|^a^	-.20	A
SC	.13	< |.10|^a^						-.17	-.27	< |.10|^a^	-.13	-.29	PP
IW	.40	-.43	< |.10|^a^						.67	.16	.52	.21	CD
A	< |.10|^a^	< |.10|^a^	< |.10|^a^	< |.10|^a^						.36	.57	.38	TRH
TH	-.13	.23	< |.10|^a^	-.32	< |.10|^a^						.44	.25	TS
CR	-.17	.15	< |.10|^a^	-.46	< |.10|^a^	.36						.35	SDL

Factors	AS	PBW	SC	IW	A	TH							

^a ^constrained to zero in the final model as correlation coefficients < |.10|. AS = access to services; PBW = physical barriers to walking; SC = street connectivity; IW = infrastructure for walking; A = aesthetics; TH = traffic hazards; CR = crime; IPW = indoor places for walking; PP = presence of people; CD = crowdedness; TRH = traffic and road hazards; TS = traffic speed; SDL = social disorder/littering.

The a priori measurement model of the NEWS-CS including only items common to the original NEWS-A met the goodness-of-fit criteria for the SRMR and RMSEA (CFI = 0.89; SRMR = 0.067; RMSEA = 0.037, 95% CI: 0.030, 0.043; χ2(217) = 358.3; AIC = -75.7). The item 'fence separating sidewalk and traffic' did not significantly load onto the factor it was supposed to measure ('Infrastructure for walking') and standardized residuals for the covariances of the items 'hilly streets', 'many dead ends', and 'major barriers (e.g., roadwork, freeways)' suggested that they were inter-correlated rather than independent (Table 4). Additionally, several latent factors were not inter-correlated. After re-defining 'fence separating sidewalk and traffic' as a single item, adding an additional factor ('Physical barriers to walking') explaining the observed inter-correlations between 'hilly streets', 'many dead ends', and 'major barriers (e.g., roadwork, freeways)', and constraining relevant inter-factor covariances to zero, the model met all goodness-of-fit criteria and yielded the lowest AIC value (CFI = 0.97; SRMR = 0.042; RMSEA = 0.021, 95% CI: 0.009, 0.029; χ2(216) = 260.7; AIC = -171.3). All standardized loadings were significant at the 0.001 level. Moderate associations were observed among 'Infrastructure for walking', 'Physical barriers to walking' and 'Access to services' and between 'Crime' and 'Infrastructure for walking' (Table 5).

## Discussion

The main aims of this study were (1) to adapt the original NEWS-A [17,18], a questionnaire gauging perceived neighborhood attributes related to walking, for Chinese seniors living in Hong Kong and similar metropolises in China; and (2) to conduct reliability and factorial validity testing of the adapted instrument NEWS-CS.

### Development of the NEWS-CS

Aspects of the built environment relevant to the target geographical setting [35] and age group [25] that were not covered by the original instrument were identified and added to the NEWS-CS. Location-specific attributes included types of food outlets (e.g., Chinese noodle shops) and other destinations (e.g., betting branches), elements of pedestrian infrastructure (e.g., pedestrian bridges and indoor places for walking), attributes associated with high levels of residential density (e.g., crowded streets) and steep terrain (e.g., slippery streets), and idiosyncratic forms of littering and social disorder (e.g., objects dropping from high-rise building). Similarly to studies conducted in the USA [11,14,35], environmental attributes identified to potentially affect walking in older residents were the presence of specific destinations (e.g., community centers and clinics), public transport infrastructure (e.g., access of shops via public transport), traffic/crime safety (e.g., parked cars blocking vision while crossing roads), and services (e.g., sitting facilities).

The original 4-point Likert scale did not require modifications as it yielded similar or higher levels of variability in responses than alternative scales. The main motivation for modifying the anchors of the Likert scale was the documented tendency of Asians to avoid extreme responses such as 'strongly agree' or 'strongly disagree', which results in restricted variability of scores [26]. No such tendency was detected in the present study. The unexpected results may be attributable to the socio-demographic characteristics of the examined sample. Namely, studies that identified low rates of extreme response styles in Asians were conducted on younger and more highly educated participants [36]. Recent findings suggest that the tendency to avoid extreme responses is negatively related to age and positively related to education [37]. This means that older respondents with a lower educational attainment would be less likely to avoid extreme responses than their younger and more highly educated counterparts.

### Reliability of the NEWS-CS

In general, the subscales and single items of the NEWS-CS showed moderate to good test-retest reliability. Unacceptably low levels of reliability were observed only for four items measuring distance to destinations. Three of these items (Westerns non-fast food restaurant and cafés; audio/video shops) pertained to services of low relevance and affordability for Hong Kong Chinese seniors. The fourth unreliable item, public toilet, could also be classified as an unlikely destination respondents would walk to from home. Given that the infrequent use of services results in poorer knowledge or awareness of their location in space [38], the low reliability indices observed for the above items are explicable. It is suggested that these four types of destination be omitted from the NEWS-CS.

Overall, the reliability of the NEWS-CS in the examined sample was lower than that reported for the original NEWS or similar instruments in adults [16,18,23,24]. Specifically, while test-retest reliability (based on ICC) of the NEWS-CS subscales ranged from 0.37 to 0.77, it ranged from 0.62 to 0.88 for the Australian version of the NEWS [23], from 0.58 to 0.80 for the original NEWS [16], and from 0.57 to 0.99 for the Chinese translation of the original NEWS-A [18]. The observed differences may be due to aging-related cognitive decline adversely affecting memory and, thus, response reliability [39]. Also, the longer interval between surveys used in this study (2-3 weeks) compared to a study on Chinese version of the NEWS-A for adults (7-10 days) might have contributed to lower levels of reliability [18]. To unravel the possible reasons for the observed discrepancies in reliability estimates, the test-retest reliability of the NEWS-CS should be examined in younger Hong Kong adults (20-65 years) and in a sample of seniors but using a shorter between-assessment interval.

### Factorial structure of the NEWS-CS

The final measurement model of the NEWS-CS encompassed 12 conceptually distinct latent factors and four single items. The model fitted the data relatively well, although one of the indices of goodness-of-fit (CFI) did not meet a more stringent criterion of model fit [33]. The CFI is sensitive to the magnitude of correlation between variables [34]. Given that co-occurring environmental characteristics are sometimes only weakly-to-moderately correlated [10], relatively robust measurement models of environmental characteristics with acceptable values for other indices (e.g., RMSEA and SRMR) may yield lower CFI values.

Contrary to expectations, the item 'fences separating sidewalk from traffic' did not load on an 'Infrastructure for walking' factor but required re-specification as a single item. The independence of similar perceived environmental features (e.g., grass/dirt strip separating sidewalks from traffic) from other elements of pedestrian infrastructure listed in the original NEWS or NEWS-A was observed in other two studies [19,21]. Overall, current cumulative evidence suggest that this item should be considered as a separate characteristic. In fact, safety fences or grass/dirt strips do not necessarily co-occur with sidewalks, cross-walks and other general pedestrian infrastructure. Another unexpected but not unique finding was the relative independence of items gauging traffic speed from items gauging traffic load and road hazards. Similar results were observed in Australia [19] but not in the USA [16,21]. In general, the roads in urban areas of Hong Kong and Australian cities have lower traffic capacity than those in the USA. It is not difficult to understand how inner-city, narrow, traffic-congested roads of Hong Kong, which are frequently affected by road construction works, could be perceived as having low-speed traffic, while more peripheral, major roads could be associated with high-speed traffic. The large variability in traffic speed across urban areas of Hong Kong may be the main reason why perceived traffic speed and load/hazards did not load on a single traffic-related factor. The third and last unexpected finding regarding the factorial structure of the NEWS-CS were the relatively high inter-correlations between items measuring physical barriers to walking, presence of dead-ends, and hilly streets, which formed a new latent factor of 'Physical barriers to walking'. These items were not correlated in three earlier studies [17,19,21]. Unlike previously examined locations (Adelaide, Seattle, and Baltimore), Hong Kong is typified by a mixture of flat and extremely hilly terrain (elevation ranging from 0 m to 958m), where dead-ends and the presence of physical barriers such as stairs or steep slopes are a common occurrence. Thus, the observed associations between these three items reflect the peculiar hilly terrain of Hong Kong.

To examine the extent to which the original measurement model of the NEWS-A could be generalized to different geographical location, culture, and age groups, confirmatory factor analysis was conducted on the items common to the NEWS-A and NEWS-CS. Similarly to the originally proposed measurement model of the NEWS-CS (see above), to achieve a good level of fit to the data, the initial model of the NEWS-A required two modifications: one related to 'fences separating sidewalks from traffic' being a single item, the other pertaining to 'hilly streets', 'dead-ends' and 'physical barriers to walking' being underlain by a common factor ('Physical barriers to walking'). As noted above, other studies found that the presence of safety fences or dirt strips separating traffic did not consistently co-occur with other perceived environmental attributes measured by the NEWS-A. Therefore, there is considerable support for it being a characteristic that is independent of other aspects of pedestrian infrastructure. The fact that the structure of other subscales of the original NEWS-A was sufficiently valid and the inter-factor correlations were similar to those observed in other samples [17,19,21] speaks in favor of the generalizability of the measurement model of the NEWS-A to geographical locations, cultures and age groups markedly different from those for which the NEWS and NEWS-A were developed [21]. The observed higher inter-correlations between three types of physical barriers to walking in Hong Kong would not constitute a problem in conducting cross-location comparative analyses on the associations between perceived environmental characteristics and physical activity behavior. This is because, in such case, these three characteristics would be modeled as three separate predictors rather than a single attribute. Notably, although correlated, these attributes were not highly collinear (the correlations ranged from 0.23 to 0.43), permitting their simultaneous inclusion in regression models.

### Limitations and future research

The main limitation of this study pertains to using a convenience sample of Hong Kong residents, all members of the Elderly Health Centres. However, the fact that previous investigations have found this particular group of seniors to be sufficiently representative of the general population is reassuring [29]. Another limitation regards the exclusive focus on Hong Kong. The use of the NEWS-CS in other Chinese and Asian urban locations may require further adaptations to capture environmental facilitators and deterrents of walking relevant to these locations. In this study, neighborhood was defined as an area within 10-15 minute walk from home. It remains to be seen whether this definition of neighborhood is clearly understood and corresponds to that of the respondents. Additionally, the effect of neighborhood size on the associations between the built environment and walking is unclear. There is evidence that different neighborhood geographic boundaries and sizes impact on the strength of associations [40]. Moreover, different definitions of neighborhood may be required to capture environmental effects on walking for different purposes. Thus, future instruments measuring perceptions of the local environment may need to use a different definition of neighborhoods for attributes associated with walking for recreation and those associated with walking for transport.

## Conclusions

The main aim of this study was to develop/adapt and validate a self-report measure of neighborhood environmental characteristics related to walking appropriate for Chinese seniors. With the exception of a few destination-related items with limited relevance to the target population, the NEWS-CS showed acceptable levels of test-retest reliability and construct validity. Importantly, the factorial structure of the items common to the original instrument (NEWS-A) corresponded to that obtained in other geographical locations, cultures and age groups [17,19,21]. These results are encouraging as they support the feasibility of cross-country and age-group comparisons of the effect of the neighborhood environment on walking. Further validation works needed to establish the generalizability of the NEWS-CS to other Asian locations and test its criterion validity, i.e., the extent to which perceived attributes of the local environment can explain walking for different purposes in Chinese seniors.

## Competing interests

The authors declare that they have no competing interests.

## Authors' contributions

EC conceptualized and coordinated the study, analyzed the data and wrote drafts of the manuscript. All authors contributed to the questionnaire adaptation. CPHS, WMC, SYH, and MCC oversaw and participated in the translation of the questionnaire. LCJL, WMC, MCC and MKT helped with the coordination of the study and data collection. All co-authors critically reviewed the drafts of the manuscript and approved its final version.
